# PP2A inhibition determines poor outcome and doxorubicin resistance in early breast cancer and its activation shows promising therapeutic effects

**DOI:** 10.18632/oncotarget.3012

**Published:** 2015-01-30

**Authors:** Raúl Rincón, Ion Cristóbal, Sandra Zazo, Oriol Arpí, Silvia Menéndez, Rebeca Manso, Ana Lluch, Pilar Eroles, Ana Rovira, Joan Albanell, Jesús García-Foncillas, Juan Madoz-Gúrpide, Federico Rojo

**Affiliations:** ^1^ Translational Oncology Division, Oncohealth Institute, Health Research Institute FJD-UAM, University Hospital “Fundación Jiménez Diaz”, Madrid, Spain; ^2^ Pathology Department, IIS “Fundación Jiménez Diaz”, E-28040 Madrid, Spain; ^3^ Institute of Health Research INCLIVA, Valencia, Spain; ^4^ Medical Oncology Department, Hospital del Mar, Barcelona, Spain

**Keywords:** PP2A inhibition, FTY720, prognosis, therapy

## Abstract

The protein phosphatase 2A (PP2A) is a key tumor suppressor which has emerged as a novel molecular target in some human cancers. Here, we show that PP2A inhibition is a common event in breast cancer and identified PP2A phosphorylation and deregulation SET and CIP2A as molecular contributing mechanisms to inactivate PP2A. Interestingly, restoration of PP2A activity after FTY720 treatment reduced cell growth, induced apoptosis and decreased AKT and ERK activation. Moreover, FTY720 led to PP2A activation then enhancing doxorubicin-induced antitumor effects both *in vitro* and *in vivo*. PP2A inhibition (CPscore: PP2A phosphorylation and/or CIP2A overexpression) was detected in 27% of cases (62/230), and associated with grade (*p* = 0.017), relapse (*p* < 0.001), negative estrogen (*p* < 0.001) and progesterone receptor expression (*p* < 0.001), HER2-positive tumors (*p* = 0.049), Ki-67 expression (*p* < 0.001), and higher AKT (*p* < 0.001) and ERK (*p* < 0.001) phosphorylation. Moreover, PP2A inhibition determined shorter overall (*p* = 0.006) and event-free survival (*p* = 0.003), and multivariate analysis confirmed its independent prognostic impact. Altogether, our results indicate that PP2A is frequently inactivated in breast cancer and determines worse outcome, and its restoration using PP2A activators represents an alternative therapeutic strategy in this disease.

## INTRODUCTION

Breast cancer is the most common diagnosed cancer and accounts for the majority of cancer-related deaths in women worldwide [1]. This is a very heterogeneous disease at both molecular and clinical levels and the conventional histopathological TNM staging criteria is still used to predict prognosis [2, 3]. Actually, breast cancer classification discriminates five different tumor subtypes and a normal breast-like group based on an immunohistochemical criteria that includes expression of estrogen (ER) and progesterone receptors (PR), and human epidermal growth factor receptor 2 (HER2). Of importance, within this classification, the triple-negative subtype includes those cases with worst prognosis and represents a major challenge from a therapeutic perspective [4]. Therefore, it remains necessary to improve our knowledge about the molecular bases that governs breast cancer pathogenesis to develop more personalized and effective therapies that enhance patient outcomes and overcome resistance to standard chemotherapy treatments [5, 6].

The protein phosphatase 2A (PP2A) is a key tumor suppressor that regulates signaling pathways with a high relevance in human cancer [7–9]. Several different molecular mechanisms to inhibit PP2A have been described in cancer cells including alterations in PP2A subunits or deregulation of endogenous PP2A inhibitors such as SET and CIP2A [10, 11]. Interestingly, downregulation and low prevalent inactivating mutations affecting PP2A have been previously reported in breast cancer [12–15]. In addition, although the role of SET in breast cancer remains mostly unknown, it has been described high SET levels and low PP2A activity in the MCF-7 cell line [16], and the work by Switzer et al. [17] showed that COG112-mediated SET inhibition in the MDA-MB-231 breast cancer cells reduced AKT signaling and cell proliferation, indicating that SET could serve as a novel therapeutic target in these cancers. Furthermore, CIP2A has been reported to be associated with breast cancer aggressivity [18] and to modulate sensitivity of breast cancer cells to bortezomib and doxorubicin treatments [19, 20]. Moreover, it has been recently reported that CIP2A deficient mice are resistant to mammary tumorigenesis, the existence of a positive feedback loop involving CIP2A and E2F1 which defines senescence sensitivity of breast cancer cells [21], and the CIP2A modulation of mTORC1 and authophagy in this disease [22]. In this study, we show that PP2A is commonly inactivated in breast cancer and identify that CIP2A and SET overexpression together with PP2A hyperphosphorylation are key contributing mechanisms to inhibit PP2A in this disease. Of importance, we show that FTY720 treatment activates PP2A through decreasing its phosphorylation and CIP2A expression and then reduces cell growth and induces caspase-dependent apoptosis. Moreover, FTY720-induced PP2A activation led to changes in AKT and ERK activation status and potentiates doxorubicin antitumor effects in both parental and mammosphere-derived breast cancer cells. To determine its clinical relevance, we quantified SET, CIP2A and PP2A phosphorylation in a series of 230 breast cancer patients, observing that PP2A inhibition is a recurrent molecular event that determines shorter overall and event-free survival. Interestingly, our results highlight that the use of PP2A activators alone or in combination with drugs such as doxorubicin could be a promising therapeutic alternative for treating an important subgroup of breast cancer patients.

## RESULTS

### Different PP2A activation levels occurs in breast cancer

**Figure 1:**
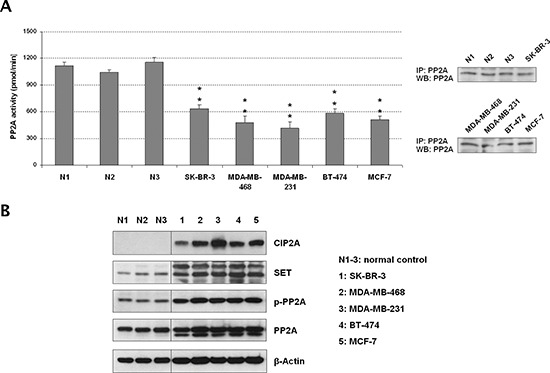
The tumor suppressor PP2A is inhibited in breast cancer cell lines **(A)** Quantification of PP2A activity and western blot showing levels of immunoprecipitated PP2A from the protein extracts used in the phosphatase assays. **(B)** Western blot analysis showing PP2A, phospho-PP2A, CIP2A and SET expression levels; *p < 0.05; **p < 0.01; N1–3: normal controls corresponding to normal mammary tissue samples obtained from Fundación Jiménez Díaz Biobank (Madrid, Spain).

We first analyzed the PP2A activation status in five cell lines representative of the different subtypes of breast cancer, observing that all of them showed a significant reduction of PP2A activity (Figure 1A). In order to investigate the molecular causes of this PP2A inhibition, we analyzed PP2A expression and phosphorylation levels on PP2A tyrosine-307 together with the expression of the endogenous PP2A inhibitors SET and CIP2A. Of importance, it has been reported that PP2Ac is inactive when tyrosine-307 is phosphorylated [23]. In concordance with the results showed by the PP2A activity assays, we found PP2A hyperphosphorylated together with SET and CIP2A markedly overexpressed in all the breast cancer cell lines analyzed (Figure 1B). To confirm their PP2A inhibitory function, CIP2A and SET depletion experiments were carried out in MDA-MB-231 and BT-474 cells, observing that both CIP2A and SET silencing led to an increased PP2A activity (Figure S1A, Figure S1B and Figure S1C). Altogether, these results would suggest that PP2A inhibition is a relevant alteration in breast cancer and indicate the simultaneous cooperation of several distinct molecular mechanisms to inhibit PP2A in this disease.

### PP2A activation by FTY720 reduces cell viability in breast cancer

To clarify the biologic relevance of PP2A deregulation in breast cancer cells, we assessed whether an increase of PP2A activity could affect their cell viability. Thus, we treated the MDA-MB-231 and BT-474 cell lines with the PP2A activator FTY720 or vehicle (DMSO). These cell lines were chosen based on their CIP2A and SET overexpression levels (Figure 1B) and because they represent aggressive breast cancer phenotypes (MDA-MB-231: triple negative; BT-474: HER-2). However, all the 5 breast cancer cell lines analyzed showed similar sensitivity to FTY720 treatment (IC50 range from 2.9 to 8.5 μM; MDA-MB-231: 2.9 μM; BT-474: 8.5 μM; MCF-7: 5.3 μM; SK-BR-3: 3.9 μM; MDA-MB-468: 4.1 μM).

**Figure 2:**
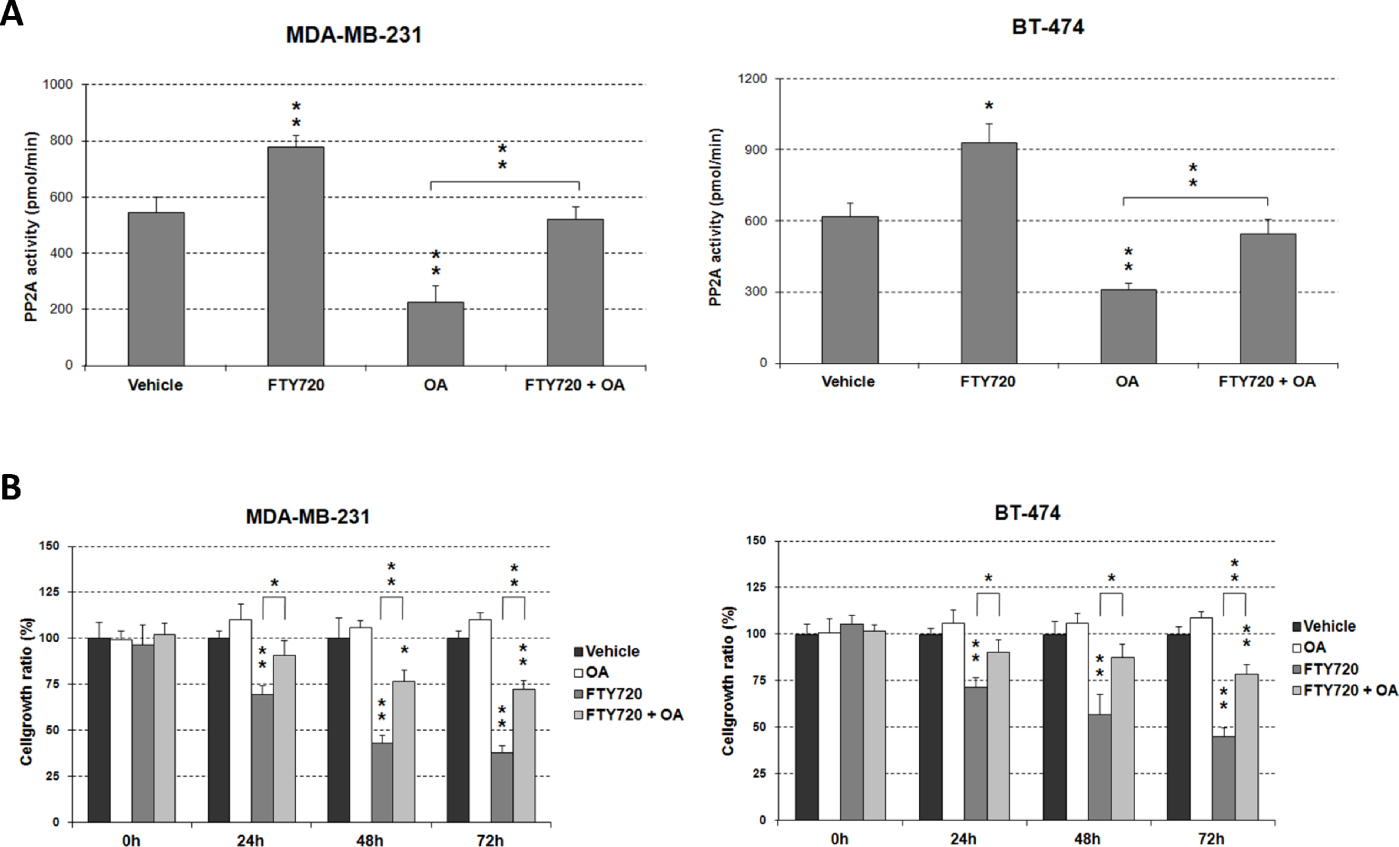
FTY720 impairs cell viability through PP2A activation **(A)** Treatment with OA inhibits the FTY720-induced PP2A activity in MDA-MB-231 and BT-474 cells. **(B)** The impaired cell growth induced by FTY720 is restored by the treatment with OA.

Furthermore, quantification of PP2A activity by phosphatase assays confirmed that FTY720 led to PP2A activation in both MDA-MB-231 and BT-474 cell lines, observing around 1.5-fold increase in the PP2A activity (Figure 2A). As a control, we pretreated MDA-MB-231 and BT-474 cells with the PP2A inhibitor OA for 90 minutes, followed by incubation with FTY720 or vehicle for 24 hours. OA was used at a concentration that inhibits PP2A but no other phosphatases [24], observing that FTY720-induced PP2A activity was inhibited by OA (Figure 2A). To evaluate whether FTY720 is a specific PP2A activator we quantified PP2A and PP1 activities in MDA-MB-231 and BT-474 cells after FTY720 treatment, observing that FTY720 was able to increase only PP2A activity. To confirm that OA is a specific PP2A inhibitor at this concentration, similar experiments were carried out (Figure S2).

To further investigate the biologic effect of the FTY720-induced PP2A activation in breast cancer, we assessed apoptosis measuring activity levels of caspase 3 and 7. In concordance with its ability to activate PP2A and decrease cell viability FTY720 induced caspase-dependent apoptosis, increasing caspase activity levels more than 5-fold in both MDA-MB-231 and BT-474 cells in comparison with vehicle-treated cells. No differences were found in vehicle-treated cells pretreated with OA. However, OA markedly reduced FTY720-induced caspase activity (Figure S3A). Altogether, these results would indicate that PP2A activation by FTY720 treatment has a promising therapeutic value in breast cancer cells.

### PP2A activation by FTY720 enhances antitumor activity of doxorubicin

**Figure 3:**
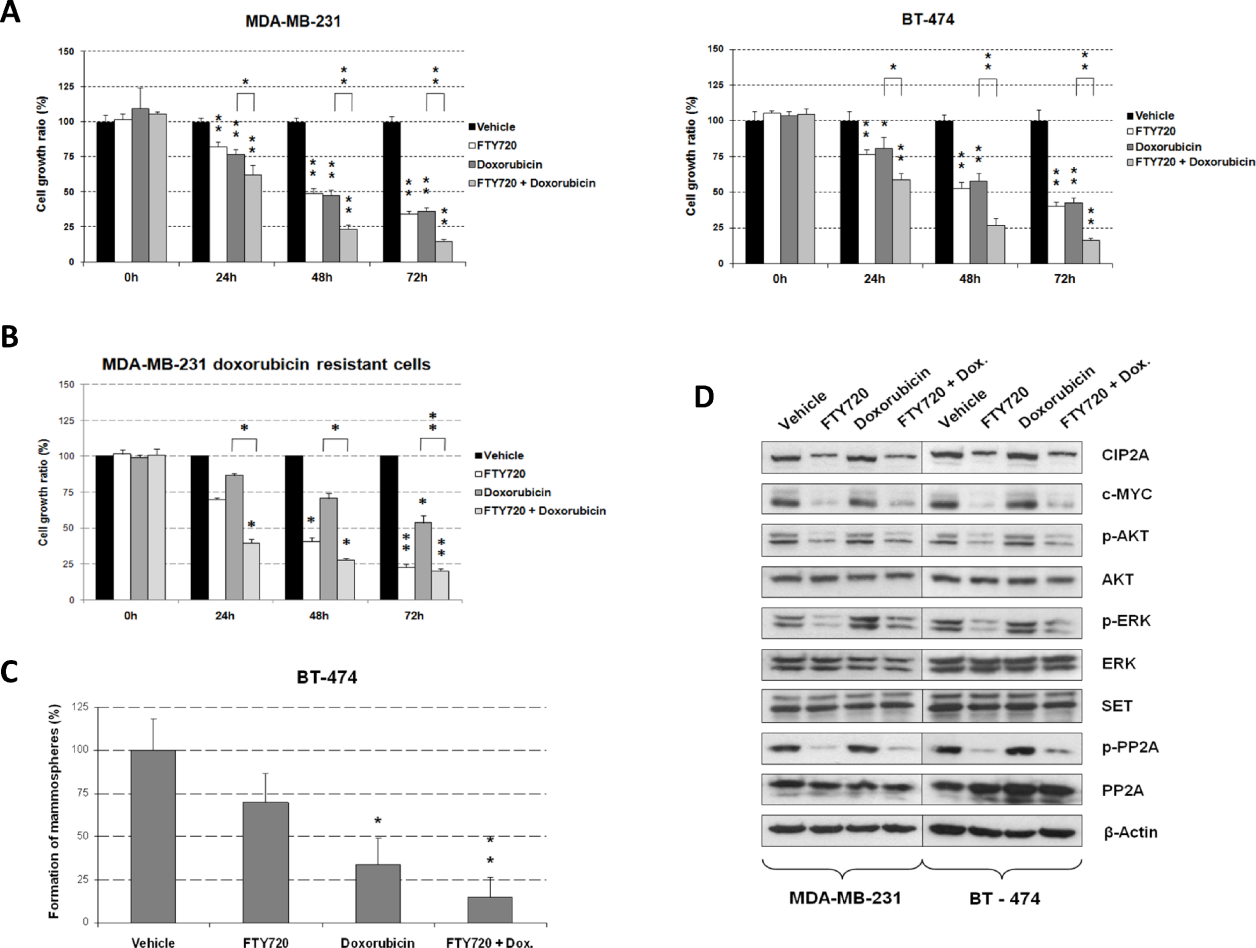
FTY720-induced PP2A activation potentiates antitumor effects of doxorubicin in breast cancer cells **(A)** MTS assays showing cell growth after FTY720 treatment in combination with doxorubicin in MDA-MB-231 and BT-474 cells. Cells treated with vehicle (DMSO) were used as controls. **(B)** MTS analysis showing that FTY720 resensitizes to doxorubicin MDA-MB-231 cells with doxorubicin acquired resistance. **(C)** Evaluation of mammosphere formation capability in BT-474 cells after doxorubicin and FTY720 treatments. **(D)** Western blot analysis showing the molecular effects induced after FTY720 treatment in combination with doxorubicin in MDA-MB-231 and BT-474 cells; *p < 0.05; **p < 0.01.

Anthracyclines like doxorubicin are among the chemotherapy drugs used in breast cancer standard systemic therapy [6]. Interestingly, we found that doxorubicin-induced antitumor effects in the MDA-MB-231 and BT-474 cell lines were markedly enhanced when cells were treated simultaneously with the PP2A activator FTY720 (Figure 3A). The microscope images obtained were in concordance with the results showed by MTS assays in both cell lines (Figure S3B). Chou-Talalay analyses showed that the FTY720/doxorubicin combination has additive effects in MDA-MB-231 cells (Combination index [CI] = 0.99), and synergistic effects in BT-474 cells (CI = 0.87). Altogether, these results show that FTY720 treatment potentiates doxorubicin-induced antitumor effects in breast cancer cells. To further confirm our hypothesis that PP2A activation sensitizes to doxorubicin treatment, we performed a genetic PP2A activation by overexpressing PP2A in MDA-MB-231 and BT-474 cells, observing that doxorubicin showed significantly enhanced antitumor effects in those cells ectopically expressing PP2A (Figure S4). Finally, we analyzed the effect of FTY720 treatment in a MDA-MB-231-derived clone with a doxorubicin resistance (in fold change compared to parental cells) of 1,92. Of importance, we observed that FTY720 was able to resensitize to doxorubicin MDA-MB-231 clones with an acquired resistance to this drug (Figure 3B).

We next evaluated mammosphere formation in BT-474 cells, observing that both FTY720 and doxorubicin decreased mammosphere formation in both number and size of mammospheres formed. However, the combination of FTY720 with doxorubicin almost completely abolished the formation of mammospheres (Figures 3C and S5A). Of interest, 10-fold lower concentration of both drugs (1 μM) was used in mammosphere formation assays since higher concentrations totally impaired mammosphere formation (Figure S5B). MDA-MB-231 cells were unable to form well-defined mammospheres. To further confirm these results on mammospheres already formed, we next performed mammosphere formation during 7 days and then treated well-defined BT-474-derived mammospheres with vehicle, FTY720 and doxorubicin alone or combined with FTY720. As expected, we found that FTY720 enhanced doxorubicin-induced antitumor effects which confirmed our previous findings (Figure S6).

Western blot analysis showed that FTY720 decreased phosphorylation of the PP2A targets AKT and ERK without affecting their expression (Figure 3C). We observed that PP2A phosphorylation was negatively affected in FTY720-treated cells compared with vehicle-treated cells. Moreover, we found reduced CIP2A levels after FTY720 treatment whereas no changes were observed in SET expression. As expected, c-MYC levels decreased in correlation with CIP2A by FTY720 (Figure 3D). We next extracted RNA from MDA-MB-231 and BT-474 cells after treatment with vehicle or FTY720 alone or combined with doxorubicin and quantified CIP2A and PP2A by real-time PCR. Interestingly, we show that CIP2A mRNA levels did not change after FTY720 treatment (Figure S7A), indicating that CIP2A downregulation is a post-translational event as well as reduced PP2A phosphorylation since both PP2A mRNA and protein expression remained without changes (Figure S7A and Figure 3D). Additionally, we have analyzed the effect of FTY720 treatment in MDA-MB-231 cells ectopically expressing CIP2A. Interestingly, we observed that CIP2A overexpression restore basal levels of c-MYC together with AKT and PP2A phosphorylation (Figure S7B). Altogether, these results would indicate that the molecular mechanism of action of FTY720 in breast cancer cells involves PP2A activation through its dephosphorylation and CIP2A downregulation together with inhibition of AKT and ERK signaling.

### *In vivo* evaluation of FTY720 alone and combined with doxorubicin shows promising antitumor effects

**Figure 4:**
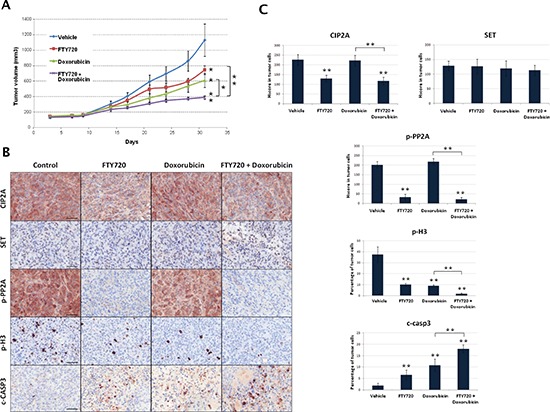
*In vivo* analysis of antitumor effects of FTY720 alone or combined with doxorubicin **(A)** Tumor growth and statistical analysis between control, FTY720, doxorubicin and FTY720+doxorubicin groups of treatment **(B)** Immunohistochemical detection of CIP2A, SET, p-PP2A, p-Histone H3 and cleaved-Caspase 3 expression in tumor samples from the different groups of treatment. **(C)** Evaluation of differential expression of CIP2A, SET, p-PP2A, p-Histone H3 and cleaved-Caspase 3.

MDA-MB-231 xenografts were performed to evaluate *in vivo* the activity of FTY720 alone or in combination with doxorubicin. We observed that tumor growth were significantly reduced by both FTY720 and doxorubicin treatments. Interestingly, FTY720 significantly enhanced doxorubicin-induced antitumor effects, confirming our previous observations *in vitro* (Figure 4A). Tumor specimens collected at the end of the experiments were analyzed by immunohistochemistry (Figure 4B). In concordance with our *in vitro* results, FTY720 led to reduced CIP2A and p-PP2A expression. As expected, SET expression was not altered in any of the treatment regimens of the *in vivo* xenograft (Figure 4C). Although the highest effects were shown by the combined treatment, both FTY720 and doxorubicin treatments significantly reduced proliferation (phosphorylated H3) and enhaced apoptosis (cleaved caspase 3) (Figure 4C).

### Prevalence of PP2A inhibition in human breast cancer and its association with molecular and phenotypic parameters

**Figure 5:**
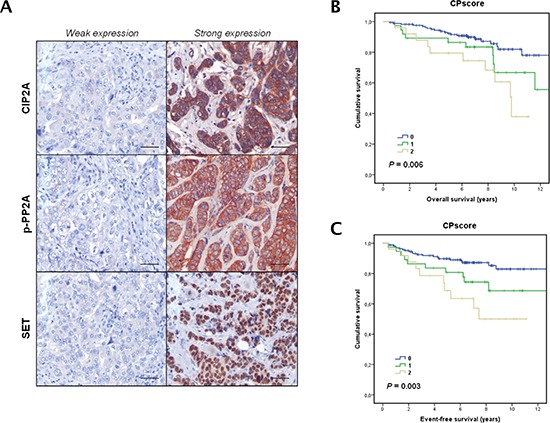
Clinical significance of PP2A phosphorylation/inhibition in breast cancer **(A)** Immunohistochemical detection of CIP2A, p-PP2A and SET showing positive and negative staining. The line shows 25 μm. Magnification x400. Kaplan-Meier analyses of overall survival **(B)** and event-free survival **(C)** in a cohort of 230 breast cancer patients.

To investigate the prevalence and clinical significance of PP2A inhibition, we quantified SET, CIP2A and phosphorylated PP2A (p-PP2A) expression in a cohort of 230 patients with early breast cancer mainly treated with anthracyclin-based adjuvant chemotherapy. Patient characteristics are presented in Table S1 and immunohistochemical detections of CIP2A, p-PP2A and SET are shown in Figure 5A. CIP2A and p-PP2A were expressed mainly in the cytoplasm of cancerous cells and diffusely distributed in the tumor. Weak staining was also detected for both markers in stromal cells (i.e. fibroblasts, lymphocytes and endothelial cells). SET was exclusively observed in malignant cells and with a predominantly nuclear location.

High p-PP2A levels were observed in 20% of cases (46/230), whereas SET and CIP2A were found overexpressed in 13.5% (31/230) and 17.8% of cases (41/230) respectively. Of relevance, SET overexpression was significantly associated and always detected in combination with high p-PP2A and/or CIP2A (*p* < 0.001; only 1 out of the 31 SET overexpressed cases was negative for p-PP2A and CIP2A) (Table S2). In addition, it was the only PP2A inhibitor marker studied without significant prognostic value in our cohort (*p* = 0.085). These observations prompted us to analyze the PP2A phosphorylation/inhibition status in breast cancer cells using a “CPscore” in which value 0 was defined by those breast cancer patients without altered p-PP2A and CIP2A, value 1 for those ones with high p-PP2A or CIP2A overexpressed, and value 2 for the subgroup of breast cancer cases with high p-PP2A and CIP2A overexpression. Thus, we observed that high CPscore associated with tumor grade (*p* = 0.017), absence of ER (*p* < 0.001) and PR expression (*p* < 0.001), and HER2 amplification (*p* = 0.049). Importantly, we also found high CPscore in those patients with higher tumor proliferation rates measured using Ki-67 (*p* < 0.001). Finally, we observed that PP2A phosphorylation/inhibition positively correlated with higher AKT (*p* < 0.001) and ERK (*p* < 0.001) phosphorylation levels in tumor cells (Figure S8A). Association between CPscore and molecular and clinical parameters are included in Table 1.

**Table 1:** Association of PP2A phosphorylation/inhibition with molecular and clinical parameters in 230 BC patients

	No. Cases	No. CPscore 0 (%)		No. CPscore 1 (%)		No. CPscore 2 (%)		*p*
CPscore	230	168	(73.0)	37	(16.1)	25	(10.9)	
T	230	168		37		25		0.552
1	113	83	(49.4)	19	(51.4)	11	(44.0)	
2	93	65	(38.7)	16	(43.2)	12	(48.0)	
3	22	19	(11.3)	1	(2.7)	2	(8.0)	
4	2	1	(0.6)	1	(2.7)	0	(0.0)	
N	230	168		37		25		0.077
0	134	104	(61.9)	17	(45.9)	13	(52.0)	
1	52	31	(18.5)	14	(37.8)	7	(28.0)	
2	26	22	(13.1)	3	(8.1)	1	(4.0)	
3	18	11	(6.5)	3	(8.1)	4	(16.0)	
Stage	228	167		37		24		0.168
1	84	68	(40.7)	9	(24.3)	7	(29.2)	
2	101	66	(39.5)	21	(56.8)	14	(58.3)	
3	43	33	(19.8)	7	(18.9)	3	(12.5)	
Grade	230	168		37		25		**0.017**
1	33	29	(17.3)	1	(2.7)	3	(12.0)	
2	108	84	(50.0)	14	(37.8)	10	(40.0)	
3	89	55	(32.7)	22	(59.5)	12	(48.0)	
Morphological type	104	83		15		6		0.754
IDC	97	76	(91.6)	15	(100.0)	6	(100.0)	
ILC	6	6	(7.2)	0	(0.0)	0	(0)	
Others	1	1	(1.2)	0	(0.0)	0	(0)	
ER	230	168		37		25		**<0.001**
Negative	90	52	(31.0)	18	(48.6)	20	(80.0)	
Positive	140	116	(69.0)	19	(51.4)	5	(20.0)	
PR	230	168		37		25		**<0.001**
Negative	107	65	(38.7)	20	(54.1)	22	(88.0)	
Positive	123	103	(61.3)	17	(45.9)	3	(12.0)	
HER2	230	168		37		25		**0.049**
Negative	157	121	(72.0)	24	(64.9)	12	(48.0)	
Positive	73	47	(28.0)	13	(35.1)	13	(52.0)	
Hormonal status	222	161		36		25		0.115
Premenopausal	59	41	(25.5)	14	(38.9)	4	(16.0)	
Postmenopausal	163	120	(74.5)	22	(61.1)	21	(84.0)	
Relapse	230	168		37		25		**<0.001**
No	158	126	(75.0)	23	(62.2)	9	(36.0)	
Yes	72	42	(25.0)	14	(37.8)	16	(64.0)	
Ki-67	230	168		37		25		**<0.001**
Low	151	123	(73.2)	14	(37.8)	14	(56.0)	
High	79	45	(26.8)	23	(62.2)	11	(44.0)	
p-ERK	211	152		34		25		**<0.001**
No	150	134	(88.2)	12	(35.3)	4	(16.0)	
Yes	61	18	(11.8)	22	(64.7)	21	(84.0)	
p-AKT	220	161		34		25		**<0.001**
No	147	128	(79.5)	16	(47.1)	3	(12.0)	
Yes	73	33	(20.5)	18	(52.9)	22	(88.0)	

IDC: invasive ductal carcinoma; ILC: invasive lobular carcinoma; ER: estrogen receptor; PR: progesterone receptor; HR: hormone receptor.

### Clinical significance of PP2A phosphorylation/inhibition in human breast cancer

Clinical follow-up data were available for all the 230 patients included in the study, with a median of age of 58 years (range: 26–90). Of relevance, high CPscore was found in those patients who relapsed (*p* < 0.001). Additionally, the subgroup of patients with high CPscore showed a substantially shorter OS (hazard ratio (HR) 1.7; 95% CI, 0.8–3.6; *p* = 0.006) and EFS (HR 1.3; 95% CI, 0.7–2.3; *p* = 0.003) (Figure 5B and Figure 5C). Moreover, we correlated CPscore with OS and EFS in those 99 breast cancer patients who received adjuvant anthracycline-based chemotherapy from our cohort of 230 cases. As expected, we observed that high CPscore was negatively associated with OS (*p* = 0.074) and EFS (*p* = 0.124). In this case, significance was not achieved probably due to the lower number of patients included in this subgroup (Figure S8B). Multivariate Cox analysis demonstrated that CPscore is an unfavorable independent factor associated with OS (HR 1.8; 95% confidence interval (CI), 1.2–2.6; *p* = 0.004) (Table 2) and EFS (HR 1.8; 95% CI, 1.2–2.7; *p* = 0.002) (Table S3) in early breast cancer.

**Table 2:** Univariate and multivariate cox analyses in the cohort of 230 BC patients (overall survival analysis)

	Univariate OS analysis			Multivariate OS Cox analysis		
	95% CI			95% CI		
	HR	Lower Upper	Significance	HR	Lower Upper	Significance
Stage			**0.003**			0.909
	1.000			1.000		
	1.951	1.262 to 3.017		0.958	0.463 to 1.985	
Grade			**0.029**			0.115
	1.000			1.000		
	1.742	1.060 to 2.864		1.565	0.896 to 2.732	
T			**0.001**			0.115
	1.000			1.000		
	1.938	1.297 to 2.897		1.637	0.887 to 3.022	
N			**<0.001**			0.055
	1.000			1.000		
	1.656	1.270 to 2.160		1.379	0.993 to 1.915	
Molecular subtype			0.813			0.327
	1.000			1.000		
	1.047	0.717 to 1.528		0.808	0.528 to 1.237	
CPscore			**0.003**			**0.003**
	1.000			1.000		
	1.768	1.221 to 2.560		1.830	1.234 to 2.716	

### PP2A phosphorylation/inhibition determines response to doxorubicin in human breast cancer patients

In order to provide clinical evidences to our hypothesis that PP2A determines doxorubicin resistance, we analyzed the PP2A inhibition status (CPscore) in an independent set of 35 patients with locally advanced breast cancer who received neoadjuvant anthracycline-based chemotherapy. Patient characteristics are shown in Table S4. The mean time from the diagnostic biopsy to the beginning of chemotherapy was 20.5 days (range 1–47 days). During that period of time, patients underwent standard clinical and radiological tumor staging. Patients received a median of 4 cycles of chemotherapy (range 2–6 cycles). All patients received an anthracycline-containing chemotherapy schedule. The overall clinical response rate (RR) (cCR and cPR) was 71.4%. After recovering from the effects of chemotherapy, the patients underwent surgery. The mean time between the last dose of chemotherapy and acquisition of the post-chemotherapy specimen from surgery was 31.2 days (range 7–61 days). Nine patients (25.7%) achieved a pathological complete response in the surgery specimen according to histopathological evaluation. Interestingly, we observed that PP2A phosphorylation/inhibition negatively correlates with both clinical (*p* = 0.01) and pathological response (*p* < 0.01) (Table S5).

To further confirm our hypothesis, we also determined CPscore, phosphorylated H3 and cleaved caspase 3 in 25 fresh breast cancer specimens treated *ex vivo* with doxorubicin. The results showed that the CPscore inversely correlated with proliferation (*p* < 0.01) and apoptosis activation (*p* < 0.01) after doxorubicin treatment (Figure S8C). Therefore, these results reinforce our clinical results regarding to the impact of the CPscore with doxorubicin resistance in breast cancer, and the potential therapeutic value of FTY720-induced PP2A activation in combination with doxorubicin in breast cancer.

## DISCUSSION

PP2A is a tumor suppressor that regulates signaling pathways crucial for maintaining tumor cell properties [7–9]. We report here that PP2A inhibition is a frequent event in breast cancer and identified PP2A hyperphosphorylation together with SET and CIP2A overexpression as molecular mechanisms that cooperate to inactivate this phosphatase. Of importance, restoration of PP2A activity after FTY720 treatment reduced cell viability, induced caspase-dependent apoptosis and inactivated AKT and ERK by decreasing their phosphorylation status. We also found that FTY720-induced PP2A activation potentiated doxorubicin antitumor effects in breast cancer cells both *in vitro* and *in vivo*. Moreover, patients with PP2A phosphorylation/inhibition had significantly worse clinical outcome and multivariate analysis, suggesting that PP2A phosphorylation/inhibition has an independent prognostic value for overall and event-free survival in breast cancer patients. Therefore, our results demonstrate that PP2A inhibition is a common event with a high clinical relevance in breast cancer and that the use of PP2A activators such as FTY720 has a potential therapeutic value and could represent an alternative therapeutic strategy for treating breast cancer patients.

Despite some data in the literature suggest a potential importance of PP2A inactivation in breast cancer, its clinical and molecular significance still need to be fully clarified. Although functional PP2A inactivating alterations have been previously reported [12–15, 25] their low prevalence suggests that this is not a key mechanism to inhibit PP2A. However, the mutational status of PP2A should be studied to evaluate their potential contribution to the reduced PP2A activity observed in breast cancer cells. Thus, we could further clarify whether the observed PP2A inhibition is the result of SET or CIP2A overexpression or inhibiting mutations of PP2A. Furthermore, several works described a potential relevant role of the PP2A inhibitor CIP2A. Thus, we observed a reduced PP2A activity in all the five breast cancer cell lines analyzed, identifying PP2A hyperphosphorylation and SET and CIP2A overexpression in all cases, which prompted us to hypothesize that these could be relevant contributing mechanisms to inhibit PP2A in breast cancer. Interestingly, PP2A mutations were not found in a recently published work after analyzing 4 breast cancer cell lines and an additional set of 25 cell lines from other tumors. Moreover, the authors studied publically available datasets (cBioPortal) and reported that PP2A activation status could be deregulated in more than 50% of basal breast cancer tumors due to alterations affecting PP2A subunits or deregulation of endogenous PP2A inhibitors such as SETBP1, SET or CIP2A [26]. However, it has been reported that SETBP1 binds and protects SET from the protease cleavage then inhibiting PP2A [27]. Therefore, it remains necessary to analyze whether SET is proteolyzed in breast cancer before concluding that SETBP1 could contribute to PP2A inhibition in this disease.

Reversible methylation of PP2A by leucine carboxyl methyltransferase 1 (LCMT-1) and protein phosphatase methylesterase 1 (PME-1) has been reported as a relevant PP2A regulatory mechanism [28]. However, their status in breast cancer still remains unexplored and further studies are needed to clarify the potential relevance of PP2A methylation in this disease. Furthermore, considering that the PP2A regulatory subunit is determining both the substrate and the location of the PP2A complex [9], it would be very interesting to study the different families of PP2A regulatory subunits to properly clarify their status and relevance in breast cancer. Moreover, future studies evaluating PP2A phosphorylation after knock-down of SET or CIP2A are warranted to assess their relevance in regulating PP2A activity in breast cancer.

FTY720 is a FDA-approved immunosuppressor used in multiple sclerosis treatment, which has shown promising preclinical effects in several human cancers [10, 29, 30]. Thus, we evaluate the potential therapeutic value of PP2A activation using FTY720 to treat breast cancer cells and observed a significant decrease of cell growth and induction of caspase-dependent apoptosis in both MDA-MB-231 and BT-474 cells, in concordance with the FTY720-mediated antitumor properties previously reported [26, 31, 32]. Importantly, we confirmed by PP2A assays that those FTY720-mediated effects were dependent on PP2A activation since pretreatment with the PP2A inhibitor OA almost totally restored these FTY720 induced effects. The fact that cell viability was not completely restored when cells were treated with FTY720 combined with OA is probably due to an additional FTY720-induced toxicity as previously reported in other tumor models such as colorectal cancer or acute myeloid leukemia [11, 30]. Therefore, the contribution of PP2A to FTY720 biological effects should be fully clarify in future studies using PP2A siRNAs or dominant active constructs. These observations are of high relevance suggesting the potential use of different PP2A activating drugs for breast cancer treatment [33].

Furthermore, FTY720 has been described to enhance the effects of sunitinib malate [34] and to increase radiation sensitivity of breast cancer cells [35]. Doxorubicin is an anthracycline currently used in breast cancer standard chemotherapy [6]. Thus, we next evaluated whether FTY720 could modulate doxorubicin sensitivity of breast cancer cells, observing that doxorubicin-induced antitumor effects were markedly enhanced after FTY720-induced PP2A activation. Moreover, we observed that FTY720 was able to resensitize to doxorubicin MDA-MB-231 clones with an acquired resistance to this drug. This observation together with the results obtained by the *ex vivo* experiments and the fact that CPscore determined response to neoadjuvant doxorubicin would indicate that PP2A activation is able to overcome doxorubicin resistance in breast cancer cells.

In addition, FTY720 led to reduced p-PP2A and CIP2A levels then enhancing PP2A activity. Of interest, CIP2A has been reported to modulate sensitivity of breast cancer cells to bortezomib and doxorubicin treatments [19, 20]. These data would indicate that the effect of FTY720 potentiating sensitivity of breast cancer cell to doxorubicin is probably via CIP2A downregulation and suggest a potential enhanced antitumor efficacy of a combination between FTY720 and bortezomib that should be explored. Moreover, CIP2A has been reported as a useful predictive marker of response to vinca alkaloid-containing chemotherapy in HER2-negative breast cancer patients [21]. Unexpectedly, a recent published work reported that FTY720 decreased sensitivity of SK-BR-3 cells to lapatinib, a compound used as HER2 targeted therapy in breast cancer. However, FTY720 was used at 2.5 μM (vs 10 μM here) during 24 hours and the authors claimed that FTY720 alone did not significantly affect the growth of the SK-BR-3 cells (2.5% growth inhibition) [36]. Besides, PP2A activity assays after FTY720 treatment are missed and one would expect lower PP2A activation with 2.5 μM than with 10 μM. Altogether, further studies to fully clarify the molecular mechanisms by with FTY720 contributes to the acquisition of a lapatinib resistant phenotype are warranted.

On the other hand, CIP2A inhibits PP2A-mediated c-MYC dephosphorylation/degradation [37], and our results show a correlation between CIP2A and c-MYC levels after FTY720 treatment. Additionally, CIP2A signature reveals the c-MYC dependency of CIP2A-regulated phenotypes and its association with breast cancer subtypes [38]. In concordance with our findings, a recent work has shown that both CIP2A and SET are frequently co-overexpressed with c-MYC in breast cancer cell lines [39]. Treatment with OP449, a novel SET inhibitor, decreases the tumorigenic potential of breast cancer cells, and SET inhibition was proposed as the best therapeutic strategy to activate PP2A – no known inhibitors of CIP2A have been described – as it could be an antitumor strategy to post-translationally target c-MYC. In contrast, we proved that extended inhibition of CIP2A expression, SET-induced PP2A inhibition and PP2A-phosphorylation by FTY720 treatment caused a deeply antitumoral effect in breast cancer cells. FTY720 binds SET and inhibits SET-PP2A interaction then leading to PP2A reactivation [40]. However, the drug does not affect SET protein expression as we observed in this work. Further, we also observed that FTY720 treatment led to reduced CIP2A expression and PP2A phosphorylation without changes in mRNA levels This post-translational FTY720 effect on CIP2A and PP2A phosphorylation should further be investigated in future works. Thus, we could better understand why this drug is a potent PP2A activator and also the potential antagonistic effects of FTY720 with other compounds as those observed with lapatinib [36] or SET-binding drugs [41].

The clinical validation in our cohort supports new therapeutic possibilities in breast cancer patients for a wide-spectrum drug like FTY720. In addition, we found reduced phosphorylation of the PP2A targets AKT and ERK after FTY720 treatment and, interestingly, we validated these results in a large series of early breast cancer patients, observing a significant correlation between PP2A phosphorylation/inhibition through the CPscore and AKT and ERK phosphorylation levels. Since SET overexpression was always detected in combination with high p-PP2A and/or CIP2A (except 1 out of the 31 SET overexpression cases) and had no significant prognostic value in our cohort, we used a CPscore in which value 0 was assigned to absence of p-PP2A and CIP2A, value 1 to high p-PP2A or CIP2A overexpression, and value 2 to those breast cancer cases with high p-PP2A and CIP2A overexpression. In fact, it has been reported higher p-PP2A in later stages of breast cancer progression and its prognostic value that was not determined [42]. Another study showed CIP2A associated with breast cancer aggressiveness [18]. PP2A has been previously reported to positively regulate expression levels of estrogen receptor alpha (ER) [43] and we observed that high CPscore significantly correlated with negative ER expression in our cohort. Importantly, absence of ER expression determines poor prognosis and lack of response to hormonal therapy and it would be indicating that PP2A inhibition confers poor outcome to breast cancer patients. Of mention, a recent work described that estradiol enhances CIP2A expression and showed higher CIP2A levels in ER-positive tissues than in ER-negative tissues [44]. These observations are not consistent with the previous observations by Keen [43] since CIP2A is a well established PP2A inhibitor. In fact, our results shows that both CPscore (as mentioned above) (*p* < 0.001) and CIP2A (*p* < 0.001) (data not shown) significantly associates with ER-negative tumors supporting that PP2A inhibition negatively correlates with ER expression. Interestingly, high CPscore was also associated with negative PR expression in our cohort which suggests a potential role of PP2A regulating PR expression that should be experimentally confirmed in future studies. Furthermore, we found a direct correlation between PP2A inhibition and HER2 positive tumors which is consistent with a previous work which described that p-PP2A is positively modulated by HER2 signaling pathway [43]. However, we unexpectedly observed that p-PP2A showed lower association with HER2 (*p* = 0.056) in our cohort than CPscore with HER2 (*p* = 0.049), and these results could be explained by a potential loop regulation between PP2A and HER2. Further studies are needed to clarify this point. Finally, to investigate the potential clinical relevance of PP2A phosphorylation/inhibition we studied a cohort of 230 breast cancer patients. Importantly, higher CPscore determined significantly shorter OS and EFS and multivariate analysis showed that PP2A phosphorylation/inhibition is a recurrent alteration with an independent prognostic value in breast cancer. However, it remains necessary to further analyze the clinical value of this CPscore in prospective studies focused on individual breast cancer molecular subtypes.

In conclusion, our results highlight that PP2A inhibition is a common event with high molecular and clinical relevance in breast cancer. Moreover, the high prevalence of this alteration suggests that PP2A hyperphosphorylation and CIP2A overexpression represent key mechanisms to inhibit PP2A in breast cancer, and our proposed CPscore would serve to define a subgroup of patients who might benefit from the inclusion of PP2A activators such as FTY720 in anticancer protocols for treating breast cancer patients alone or combined with an anthracycline-based chemotherapy backbones.

## METHODS

### Cell cultures

MDA-MB-231 (ATCC HTB-26), MDA-MB-468 (ATCC HTB-132) and SK-BR-3 (ATCC HTB-30) were cultured in DMEM/F12 (Sigma Aldrich) with 10% fetal bovine serum (FBS) (Life technologies); MCF-7 (ATCC HTB-22) in DMEM with 10% FBS; and BT-474 (ATCC HTB-20) in DMEM/F12 with 10% FBS and insulin (0.01 μg/ml). Cell lines were grown as monolayers at 37°C in a 5% CO2 atmosphere. Media were supplemented with L-glutamine (2 nM) (Gibco), penicillin G (100 U/ml), and streptomycin (100 μg/ml) (Gibco). Cells were purchased from American Type Culture Collection (ATCC) and authenticated (LGC Standards). Reagents: doxorubicin (10 μM) (Sigma Aldrich), okadaic acid (OA) (2.5 nM) and FTY720 (10 μM) (Calbiochem). To generate doxorubicin-resistant cells, MDA-MB-231 cells were cultured in the presence of increasing doses of doxorubicin (three subculturing cycles per concentration), starting at 0.5 μM. In order to assess the evolution of resistance, we determined the IC50 after every doxorubicin concentration point, by using an MTS assay (Promega) at 24 h of treatment. The resistance of every doxorubicin-resistant clone was defined as the ratio between resistant and parental cells IC50 values.

### Patient samples

Surgical resection specimens from primary breast tumors were obtained from Parc de Salut Mar Biobank (MARBiobanc, Barcelona, Spain), Fundación Jiménez Díaz Biobank (Madrid, Spain) and Valencia Clinic Hospital Biobank (Valencia, Spain). Tumor specimens from formalin-fixed paraffin-embedded (FFPE) blocks were retrospectively selected from consecutive breast cancer patients diagnosed between 1998 and 2000, which had the following criteria: infiltrating carcinomas, operable, no neoadjuvant therapy, enough available tissue and clinical follow-up. TNM (tumor–node–metastasis) staging was classified using the American Joint Committee on Cancer (AJCC) staging system. Histological grade was defined according Scarff–Bloom–Richardson modified by Elston criteria [45]. ER and PR were determined by immunohistochemistry (IHC) (SP1 and PgR636 clones, respectively; Dako, Carpinteria, CA) establishing positivity criteria in > 1% of nuclear tumor staining [46]. HER2 amplification was assayed by FISH (Pathvysion; Abbott Laboratories, Abbott Park, IL) [47]. Ki-67 was studied by IHC (MIB1 clone; Dako) [48]. Clinical data were collected from medical clinical records by oncologists. Representative areas of each tumor were carefully selected and three tissue cores (1 mm diameter) were obtained using a TMA workstation (T1000 Chemicon).

In addition, we included in the study an independent series of 35 patients with locally advanced breast cancer who had been treated with neoadjuvant chemotherapy from FJD-Biobank and pre-chemotherapy diagnostic core needle biopsies were achieved. Pre-treatment tumor specimens were histologically evaluated. As a part of the established routine protocol, ER, PR, HER2 and Ki67 were evaluated by IHC and HER2 amplification was determined by FISH. High proliferation in breast cancer based on Ki67 labelling by IHC was defined following the 13^th^ St Gallen International Breast Cancer Conference (2013) criteria based on a threshold ≥ 20% of proliferation [49]. Clinical data and follow-up were obtained from review of the patients' medical records. Pre-treatment patient staging was classified using the American Joint Committee on Cancer (AJCC) staging system for breast cancer. Clinical tumor response to primary chemotherapy was evaluated according to the International Union Against Cancer Criteria [50]. A clinical complete response (cCR) was defined as the disappearance of all detectable malignant disease within the breast by physical examination. A reduction greater than 50% in the product of the two maximum perpendicular diameters of the tumor was classified as clinical partial response (cPR). Clinical progressive disease (cPD) was considered as an increase of at least 25%. Clinical stable disease (cSD) was defined when clinical breast cancer response did not meet the criteria for cCR, cPR or cPD. Post-chemotherapy specimens were evaluated for pathological response. A pathological complete response (pCR) was defined as no histological evidence of invasive disease in the tumor specimen [51].

### *Ex vivo* models

Tissue slices larger than 1.5 cm from fresh surgical specimens of patients newly diagnosed with invasive breast cancer were obtained to add *ex vivo* doxorubicin and assess molecular effects [52]. The samples were processed in sterile conditions immediately after surgical resection. Incubation was performed in 24-well plates at 37°C in a constant atmosphere of 5% CO2 for 24 hours. At 24 hours, the specimens were fixed in 10% neutral-buffered formalin for 16 hours at room temperature and embedded in paraffin under vacuum conditions. These specimens were assayed for molecular markers as described in the IHC section.

### Western blot analysis

Protein extracts were isolated using TRIzol Reagent (Invitrogen) following manufacturer's indications, clarified (12,000xg, 15min, 4°C), denatured and subjected to SDS-PAGE and Western-blot. Antibodies: rabbit polyclonal anti-SET (Abcam), rabbit monoclonal anti-PP2AY307 (Epitomics), rabbit polyclonal anti-AKT, rabbit polyclonal anti-ERK1/2 (Cell Signaling Technology), rabbit polyclonal anti-pAKT, rabbit poly-clonal anti-pERK1/2 (Santa Cruz Biotechnology), rabbit polyclonal anti-CIP2A, and mouse monoclonal anti-β-Actin (Sigma). Proteins were detected with the appropriate secondary antibodies conjugated to alkaline phospatase (Sigma) by chemiluminescence using Tropix CSPD and Tropix Nitro Block II (Applied Biosystems).

### Proliferation assay and cell viability

Cell proliferation was measured in triplicate wells by MTS assay in 96-well plates using the CellTiter 96 AQueous One Solution Cell Proliferation Assay (Promega), following the manufacturer's indications.

### PP2A phosphatase activity assays

Protein extracts were isolated using TRIzol Reagent (Invitrogen). PP2A assays were performed with 50 μg protein extracts using a PP2A immunoprecipitation phosphatase assay kit (Millipore) and following the manufacturer's instructions. Briefly, PP2A was immunoprecipitated using 4 μg of PP2A antibody and 25 μl Protein A agarose slurry, both supplied by the kit. After 2 h of incubation in constant rocking, samples were washed 3 times with TBS 1X followed by one additional wash with a ser/thr assay buffer also provided by the kit. Next, 60 μl of a diluted phosphopeptide at 750 μM and 40 μl of ser/thr assay buffer were added, and the mix was incubated for 5 min at 30°C in a shaking incubator, and then 25 μl of the mix was transferred into each well of a 96-well plate. Each measurement was performed in triplicates. 100 μl of Malachite Green Detection Solution was added, and the mix was incubated for 15 min at room temperature. Absorbance at 650 nm was used to calculate the amount of phosphate released (pmol) using a standard curve (0–2000 pmol).

### Analysis of caspase activation

Quantification of caspase 3/7 activities was carried out using the caspase Glo-3/7 assay kit (Promega), following the manufacturer's indications. Differences in caspase-3/7 activity are expressed as fold-change in luminescence.

### Mammospheres

For the generation of mammospheres, 10000 cells were plated in 6-well ultra-low attachment plates (Corning). BCCL cells were grown in serum-free medium DMEM/F12+GlutMAXTM-I (Gibco) containing 1% N2 (Gibco), 2% B27 (Gibco), 20 ng/ml human FGF (Sigma) and 50 ng/ml EGF (Sigma). After 7 days, plates were analyzed for mammosphere formation.

### Quantitative real-time RT-PCR

Total RNA extracts were isolated with TRIzol Reagent (Invitrogen) following manufacturer's indications. RNA purity and integrity were assessed by spectrophotometric determination (NanoDrop ND-2000, NanoDrop Technologies, USA). Then, RNA was reversely transcribed to cDNA using Transcriptor Universal cDNA Master (Roche Life Science). cDNA amplification was done in a 7500 Fast Real-Time PCR System (Applied Biosystems) at 40 cycles, and using TaqMan Gene Expression Assays specific for CIP2A and PP2A (Applied Biosystems). GAPDH was used as internal control. Relative gene expression was calculated according to the comparative cycle threshold (Ct) method.

### *In vivo* animal model

All animal work was conducted as per the Barcelona Biomedical Research Park (PRBB) Institutional Animal Care and Scientific Committee guidelines. Briefly, 46-week old female Balb/C nude mice were subcutaneously inoculated in their flank with 10 × 10^6^ MDA-MB-231 cells mixed with Matrigel as previously described [53]. Tumor growth was measured twice a week. Mice bearing subcutaneous 100–150 mm^3^ tumors were distributed homogenously into four groups of 10 mice each. The first group received saline vehicle intraperitoneally (i.p.) with no active drugs. In the second group, doxorubicin (2 mg/kg in saline) was inoculated i.p. twice a week [54]. In the third group, FTY720 (10 mg/kg in saline) was inoculated i.p. every two days [31]. The last group received the combination of both drugs at the same doses. At the end of the experiment, tumors were harvested and formalin-fixed.

### Immunohistochemistry

Immunostainings were performed on tissue sections (3 μm) obtained from FFPE tumors as previously described [55]. Briefly, heat antigen retrieval was performed in pH9 EDTA-based buffer (Dako) and slides were incubated with same primary antibodies against CIP2A, SET p-PP2A, p-AKT or p-ERK [50], followed of appropriate anti-Ig horseradish peroxidase-conjugated polymer (Flex+, Dako). Sections were visualized with 3,3′-diaminobenzidine as a chromogen. All stainings were performed in a Dako Autostainer. Sections incubated with normal non-immunized rabbit immunoglobulins were used as negative controls. As positive control, sections of breast tumor with known expression of targets were used. Antibody sensitivity was calculated in a range of crescent dilutions of primary antibody (CIP2A: 1:20–1:200, SET: 1:100–1:5000, p-PP2A: 1:200–1:2000, p-AKT: 1:1–1:100, p-ERK: 1:50–1:500). Specificity was confirmed in a set of paired fresh frozen and FFPE samples were processed by western blot and IHC. Antigen preservation in tissues was confirmed assaying for expression of phospho-tyrosines using a monoclonal antibody to tyrosine-phosphorylated proteins (clone 4G10, 1:500, Millipore). Only the membrane of epithelial cells, but not stromal cells, was evaluated for CIP2A, SET and p-PP2A expression blinded to clinical data was evaluated by two investigators (FR and SZ). A semiquantitative histoscore (Hscore) was calculated by estimation of the percentage of tumor cells positively stained with low, medium, or high staining intensity. The final score was determined after applying a weighting factor to each estimate. The formula used was Hscore = (low %) × 1 + (medium %) × 2 + (high %) × 3, and the results ranged from 0 to 300.

### Statistical analysis

Statistical analyses were performed using SPSS 20 for windows (SPSS Inc, Chicago Illinois). Overall survival (OS) was defined as the time from diagnosis to the date of death from any cause or last follow-up. Event-free survival (EFS) was defined as the time from diagnosis until first event, in which relapse at any location, death or end of follow-up were considered events. Kaplan-Meier method and survival comparisons were done with the log-rank test. The Cox proportional hazards model was adjusted taking into consideration significant parameters in univariate analysis. A *p*-value less than 0.05 was considered statistically significant. Receiver operating curve (ROC) was used to determine the optimal cutoff point based on progression end point for p-PP2A, SET and CIP2A expression as previously described [56]. This work was carried out in accordance with Reporting Recommendations for Tumor Marker Prognostic Studies (REMARK) guidelines [57]. Analysis of experimental conditions was done by paired *t*-test. All the statistical tests were conducted at two sided 0.05 level of significance. Chou-Talalay analysis was performed using the CompuSyn Software (ComboSyn, Inc) and CI was used to determine additive/synergism calculations between FTY720 and doxorubicin treatments. CI< 0.90, 0.90 < CI < 1.10 and CI> 1.10 were considered, respectively, synergistic, additive and antagonistic effects.

## SUPPLEMENTARY FIGURES AND TABLES


