# Supplementary material for: PP2A inhibition determines poor outcome and doxorubicin resistance in early breast cancer and its activation shows promising therapeutic effects
Source: Oncotarget. 2015 Jan 30;6(6):4299–314. doi: 10.18632/oncotarget.3012 (PMC4414191; doi:10.18632/oncotarget.3012)
Supplement: Supplementary file 1 [file oncotarget-06-4299-s001.pdf]

SUPPLEMENTARY FIGURES AND TABLES

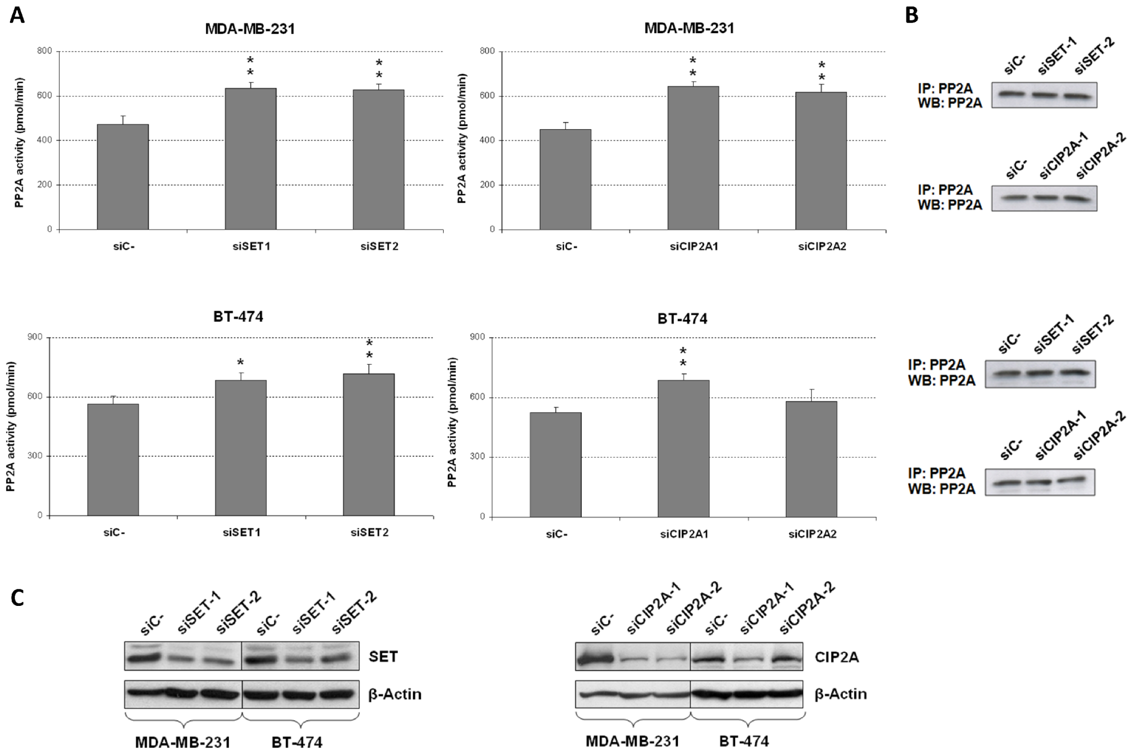

Supplementary Figure S1: Analysis of PP2A activity after depletion of SET or CIP2A. (A) Quantification of PP2A activity in MDA-MB-231 and BT-474 after SET or CIP2A silencing. **(B)** Levels of immunoprecipitated PP2A from the protein extracts used in the phosphatase assays. **(C)** Validation of SET and CIP2A silencing by western blot.

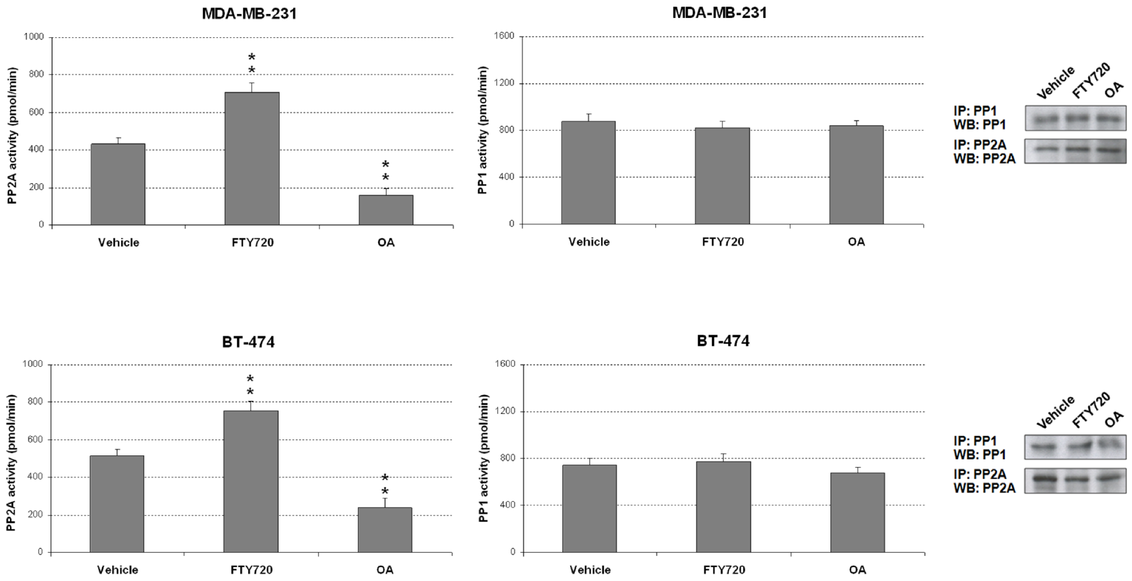

Supplementary Figure S2: Quantification of PP2A and PP1 activities in MDA-MB-231 and BT-474 after FTY720 or OA treatments. Levels of immunoprecipitated PP2A and PP1 are shown.

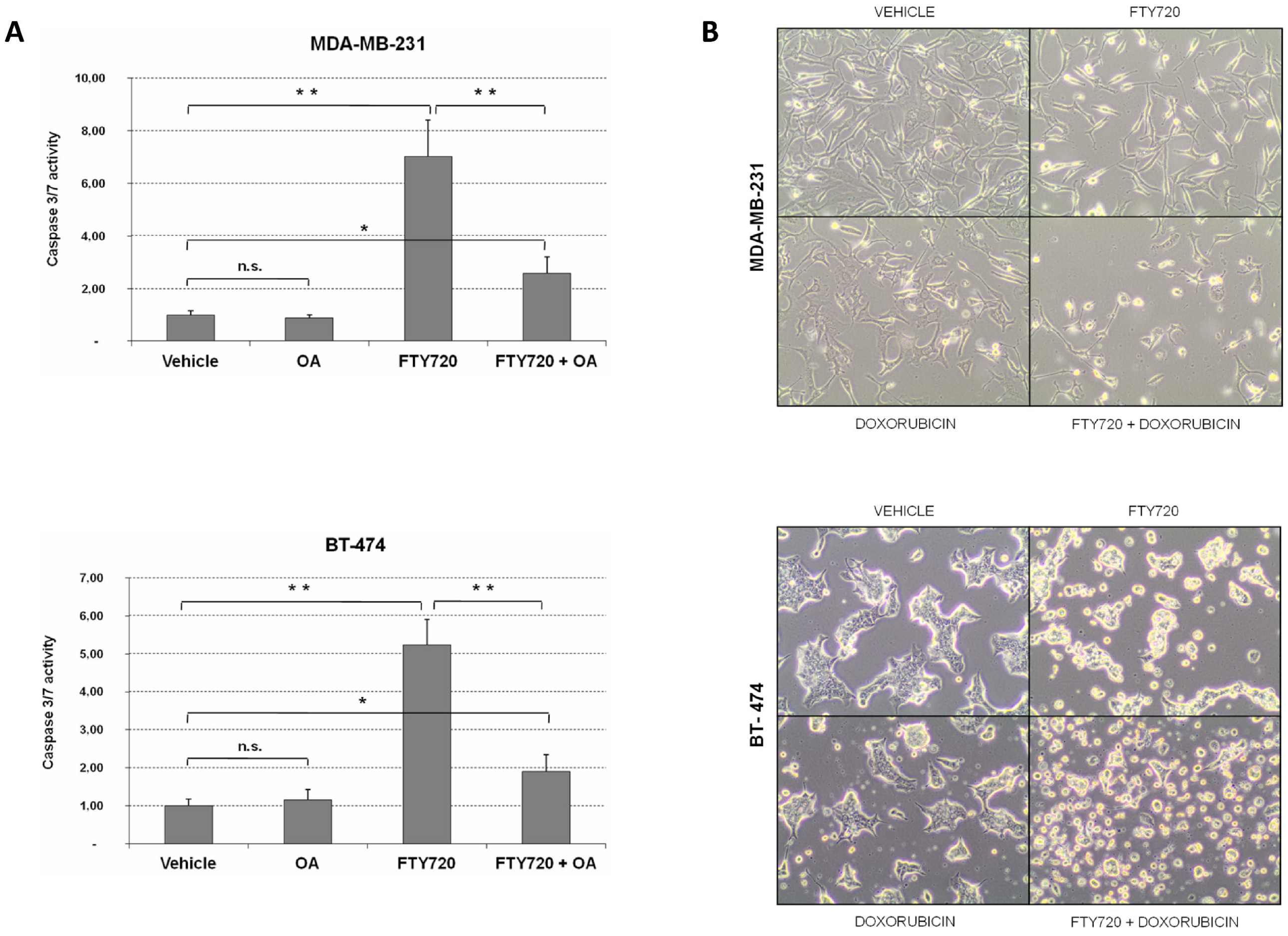

Supplementary Figure S3: FTY720-induced effects in breast cancer cells. **(A)** Caspase 3/7 assays in MDA-MB-231 and BT-474 cells after FTY720 treatment alone or in combination with OA. **(B)** Optical microscope images showing changes in morphology and cell viability after FTY720 treatment in MDA-MB-231 and BT-474 cells.

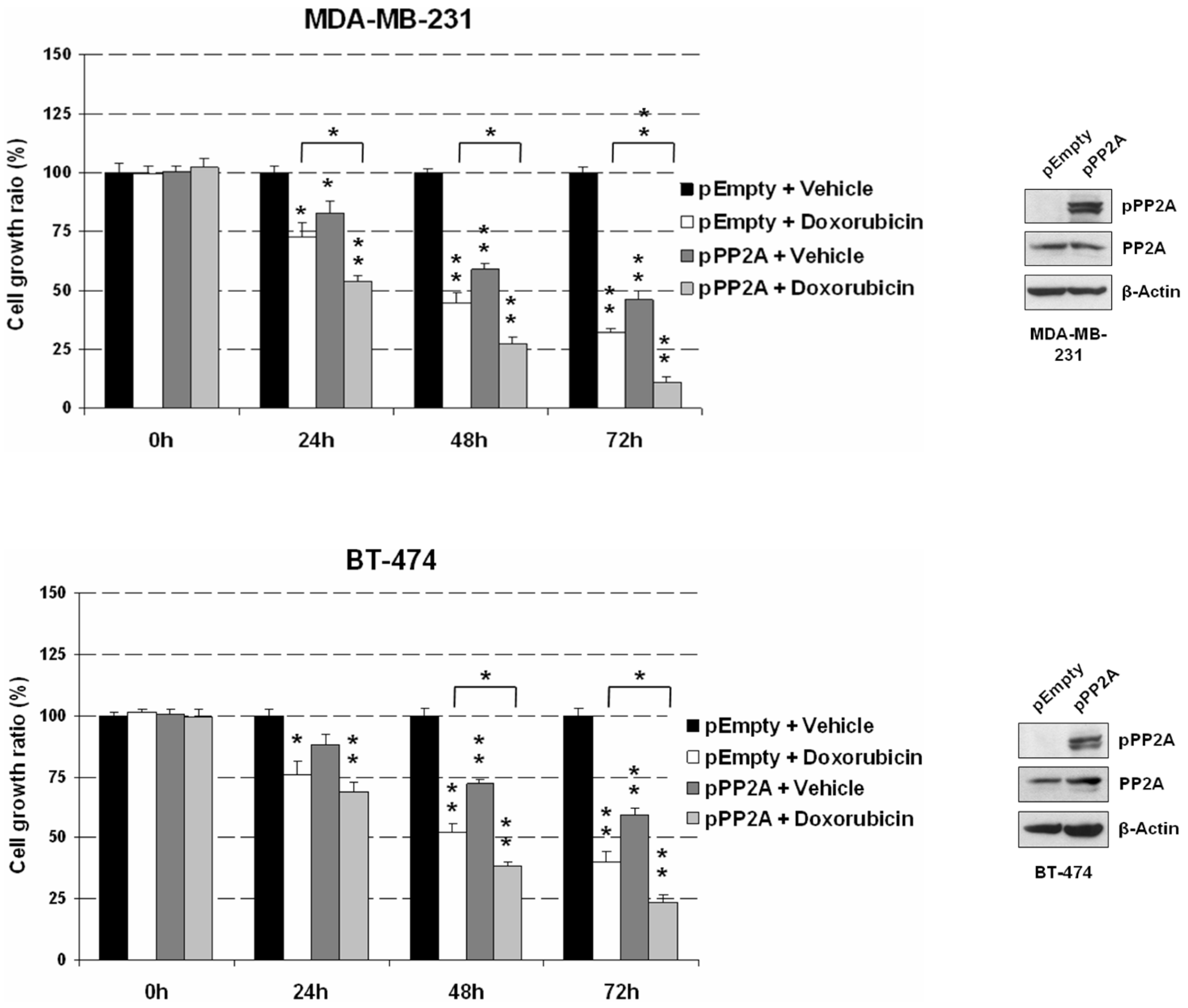

Supplementary Figure S4: PP2A overexpression potentiates antitumor effects of doxorubicin in breast cancer cells. (A) MTS assays showing cell growth after FTY720 treatment in MDA-MB-231 and BT-474 cells trandfected with PP2A or with an empty vector as control; $*p < 0.05$; $**p < 0.01$.

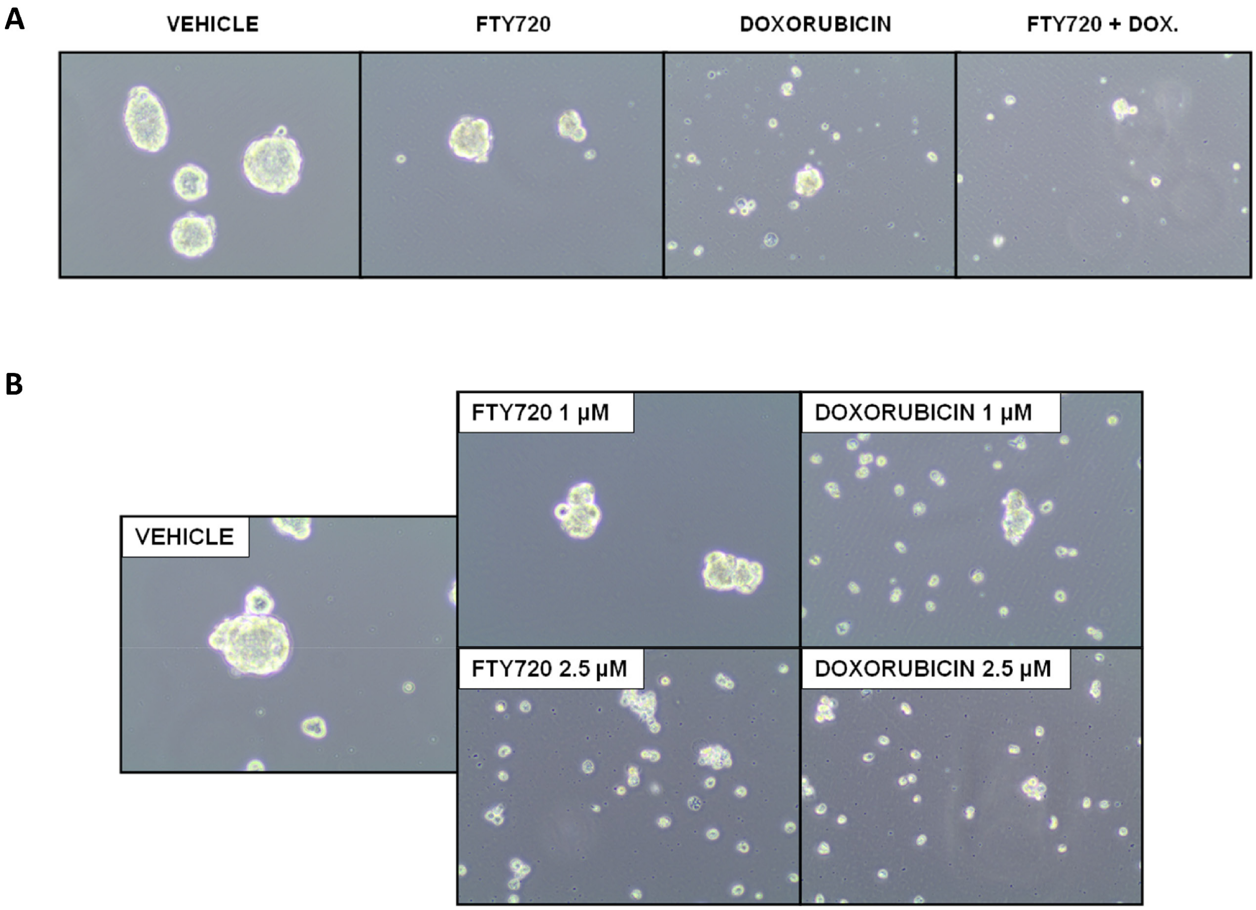

Supplementary Figure S5: Doxorubicin and FTY720 treatments in mammosphere-derived from BT-474 cells. (A) Optical microscope images showing antitumor effects of doxorubicin (1 µM) and FTY720 (1 µM) treatments alone or combined in mammospheres derived from BT-474 cells. **(B)** Optical microscope images showing effects of FTY720 or doxorubicin treatments at higher concentrations in the formation of BT-474-derived mammospheres.

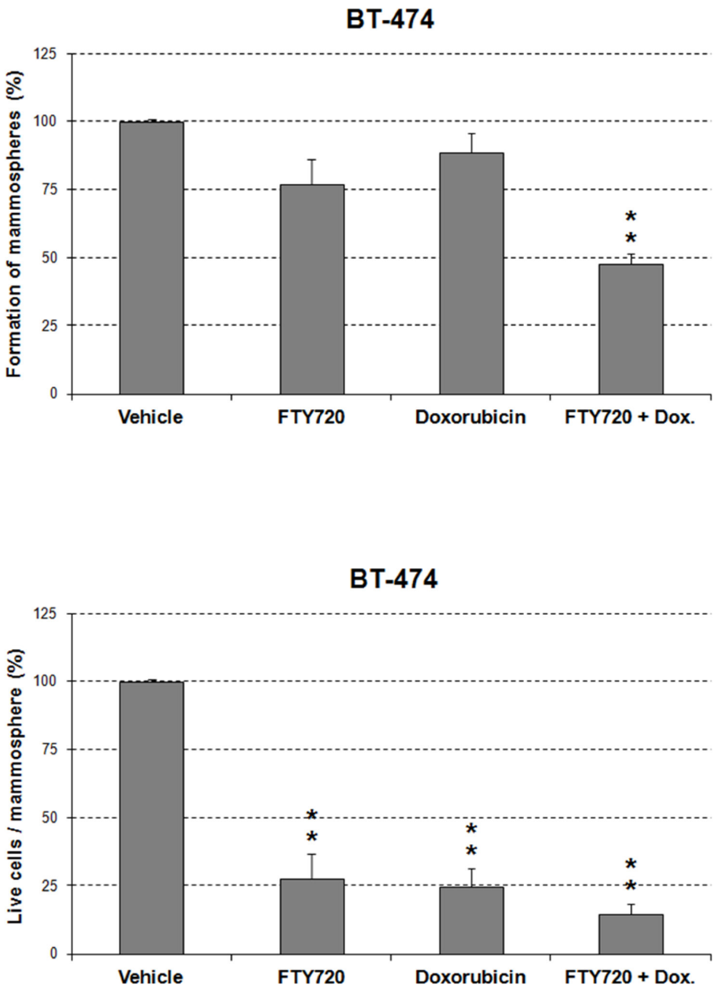

Supplementary Figure S6: FTY720 potentiates doxorubicin-induced antitumor effects in both number and size of previously formed BT-474-derived mammospheres.

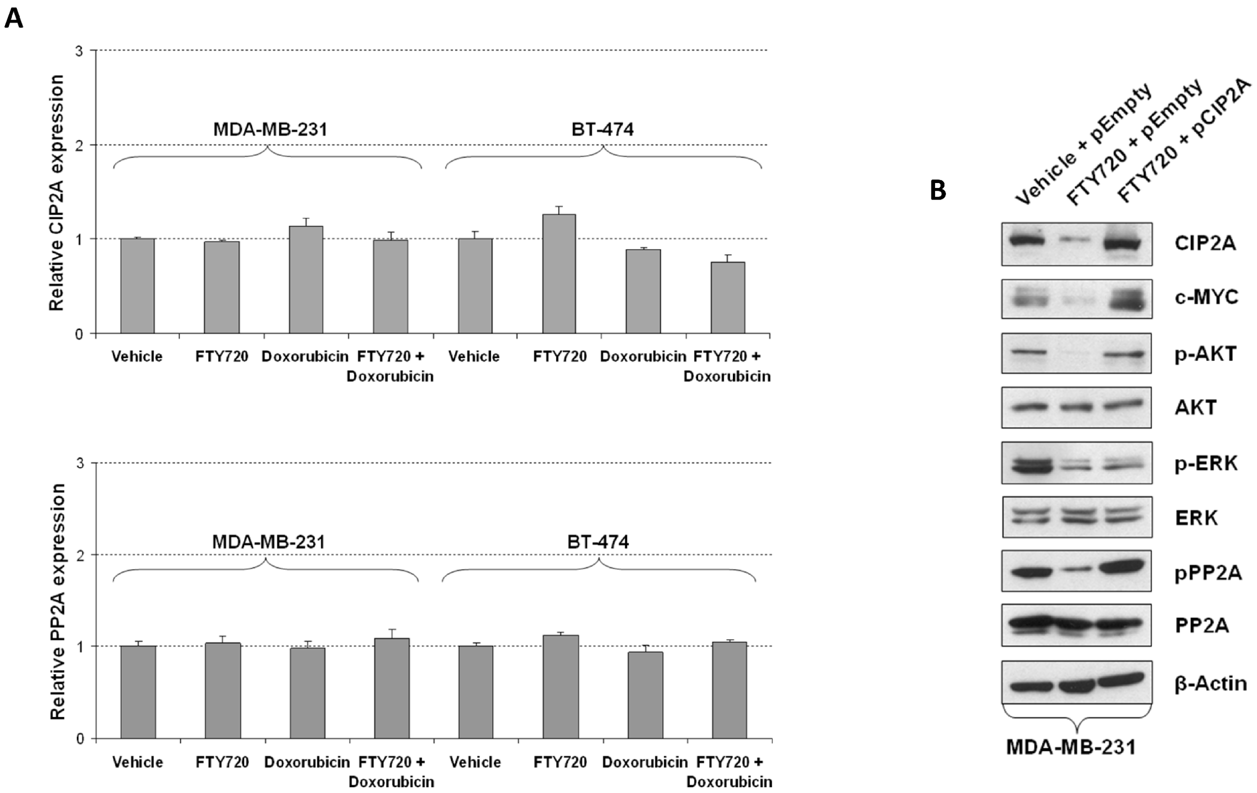

Supplementary Figure S7: (A) Quantification of CIP2A and PP2A by real-time PCR in MDA-MB-231 and BT-474 cells after treatment with vehicle, FTY720 and doxorubicin alone or combined with FTY720. **(B)** Western blot analysis showing the effect of FTY720 treatment in cells ectopically expressing CIP2A.

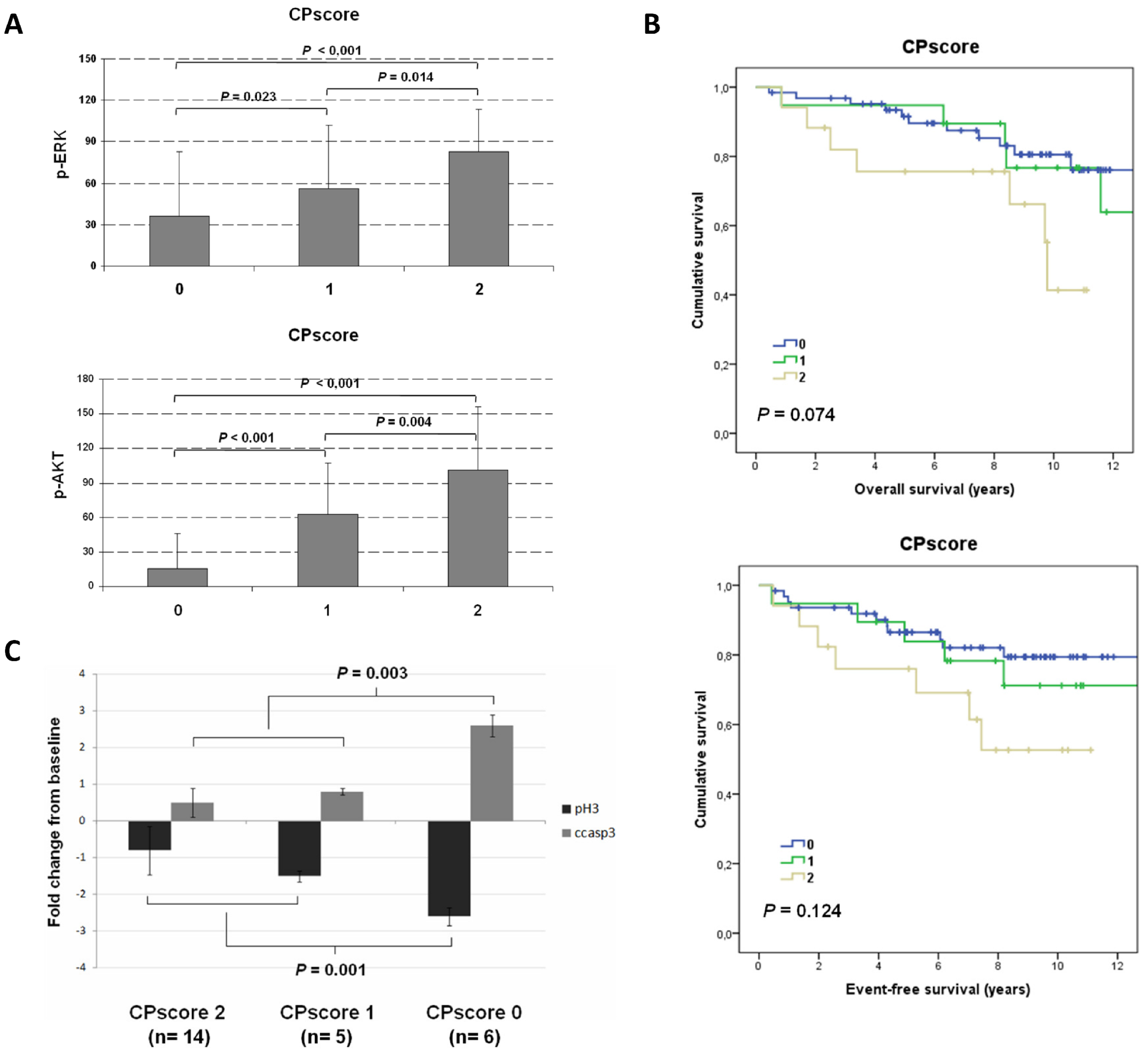

Supplementary Figure S8: Clinical significance of CPscore in breast cancer. **(A)** Correlation between the CPscore and p-ERK and p-AKT intensity defined by the histoscore values. **(B)** Kaplan-Meier analyses of overall and event-free survival in those cases who received adjuvant anthracycline-based chemotherapy (*n* = 99). **(C)** Correlation between CPscore, phosphorylated H3 and cleaved caspase 3 in 25 fresh breast cancer specimens treated *ex vivo* with doxorubicin; pH3: phosphorylated Histone H3; ccasp3: cleaved-Caspase 3.

Supplementary Table S1: Clinical and molecular characteristics of a series of 230 patients with early breast cancer

		No. (%)	
Age			
	58 (26–90)		
T			
	1	113	(49.1)
	2	93	(40.4)
	3	22	(9.6)
	4	2	(0.9)
N			
	0	134	(58.3)
	1	52	(22.6)
	2	26	(11.3)
	3	18	(7.8)
Stage			
	1	84	(36.8)
	2	101	(44.3)
	3	43	(18.9)
Grade			
	1	33	(14.3)
	2	108	(47.0)
	3	89	(38.7)
Morphological type			
	IDC	97	(93.3)
	ILC	6	(5.8)
	Others	1	(1.0)
ER			
	Negative	90	(39.1)
	Positive	140	(60.9)
PR			
	Negative	107	(46.5)
	Positive	123	(53.5)
HER2			
	Negative	157	(68.3)
	Positive	73	(31.7)
Hormonal status			
	Premenopausal	59	(26.6)
	Postmenopausal	163	(73.4)

(*Continued*)

		No. (%)	
Chemotherapy			
	None	53	(23.0)
	Adjuvant	154	(67.0)
	Neoadjuvant	23	(10.0)
Hormone therapy			
	No	84	(38.5)
	Yes	134	(134)
Relapse			
	No	158	(68.7)
	Yes	72	(31.3)
Ki-67			
	Low	151	(65.7)
	High	79	(34.3)
Molecular subtype			
	Luminal	96	(41.7)
	HER2-positive	73	(31.7)
	Triple-negative	61	(26.5)

Supplementary Table S2: Association between SET and CIP2A or p-PP2A in 230 patients with breast cancer

	No. Cases	No. SET− (%)		No. SET+ (%)		*p*
SET	230	199	(86.5)	31	(13.5)	
CIP2A	230	199		31		**<0.001**
Negative	189	177	(88.9)	12	(38.7)	
Positive	41	22	(11.1)	19	(61.3)	
p-PP2A	230	199		31		**<0.001**
Negative	184	176	(88.4)	8	(25.8)	
Positive	46	23	(11.6)	23	(74.2)	

Supplementary Table S3: Univariate and multivariate Cox analyses in the cohort of 230 BC patients (event-free survival analysis)

	Univariate EFS analysis			Multivariate EFS Cox analysis			
		95% CI				95% CI	
	HR	Lower Upper	Significance	HR	Lower Upper		Significance
Stage			**0.001**				0.691
	1.000			1.000			
	2.009	1.305 to 3.094		0.858	0.405 to 1.820		
Grade			**0.029**				0.209
	1.000			1.000			
	1.729	1.059 to 2.825		1.406	0.826 to 2.395		
T			**0.001**				0.060
	1.000			1.000			
	1.999	1.347 to 2.967		1.789	0.976 to 3.277		
N			**<0.001**				**0.022**
	1.000			1.000			
	1.727	1.324 to 2.253		1.489	1.060 to 2.092		
CPscore			**0.001**				**0.002**
	1.000			1.000			
	1.849	1.281 to 2.667		1.817	1.245 to 2.653		

Supplementary Table S4: Clinical and molecular characteristics of 35 locally advanced breast cancer patients who received neoadjuvant anthracycline-based chemotherapy

		No. (%)	
**Age**			
	59 (30–77)		
**T**			
	2	5	(14.3)
	3	30	(85.7)
**N**			
	0	29	(82.9)
	1	5	(14.2)
	2	1	(2.9)
**Grade**			
	1	3	(8.6)
	2	24	(68.5)
	3	8	(22.9)
**Morphological type**			
	IDC	34	(97.1)
	ILC	1	(2.9)
**ER**			
	Negative	7	(20.0)
	Positive	28	(80.0)
**PR**			
	Negative	6	(17.1)
	Positive	29	(82.9)
**HER2**			
	Negative	35	(100)
	Positive	0	0
**Hormonal status**			
	Premenopausal	6	(17.1)
	Postmenopausal	29	(82.9)
**Ki-67**			
	Low	26	(74.3)
	High	9	(25.7)

(*Continued*)

		No. (%)	
**Neoadjuvant chemotherapy regimen**			
	Anthracyclin-containing	35	(100)
**Clinical response**			
	Complete response	12	(34.3)
	Partial response	13	(37.1)
	Stable disease	10	(28.6)
**Pathological response**			
	Complete response	9	(25.7)

Supplementary Table S5: CPscore in locally advanced breast cancer patients who received neoadjuvant anthracycline-based chemotherapy

	CPscore		
	**0**	**1**	**2**
Pre-chemotherapy specimens (n = 35)	(n = 19)	(n = 6)	(n = 10)
Clinical response	17	2	6
No clinical response	2	4	4
Overall response rate	89.5%	33.3%	60%
***P*-value (0 vs 1 + 2)**		**0.011**	
Pathological complete response	8	0	1
No pathological complete response	11	6	9
Overall response rate	36.8%	0%	10%
***P*-value (0 vs 1 + 2)**		**0.009**	
